# Host Epigenetics in Intracellular Pathogen Infections

**DOI:** 10.3390/ijms21134573

**Published:** 2020-06-27

**Authors:** Marek Fol, Marcin Włodarczyk, Magdalena Druszczyńska

**Affiliations:** Division of Cellular Immunology, Department of Immunology and Infectious Biology, Faculty of Biology and Environmental Protection, University of Lodz, 90-237 Lodz, Poland; marcin.wlodarczyk@biol.uni.lodz.pl (M.W.); magdalena.druszczynska@biol.uni.lodz.pl (M.D.)

**Keywords:** epigenetic modifications, intracellular pathogens, immune cells

## Abstract

Some intracellular pathogens are able to avoid the defense mechanisms contributing to host epigenetic modifications. These changes trigger alterations tothe chromatin structure and on the transcriptional level of genes involved in the pathogenesis of many bacterial diseases. In this way, pathogens manipulate the host cell for their own survival. The better understanding of epigenetic consequences in bacterial infection may open the door for designing new vaccine approaches and therapeutic implications. This article characterizes selected intracellular bacterial pathogens, including *Mycobacterium* spp., *Listeria* spp., *Chlamydia* spp., *Mycoplasma* spp., *Rickettsia* spp., *Legionella* spp. and *Yersinia* spp., which can modulate and reprogram of defense genes in host innate immune cells.

## 1. Introduction

**Figure 1:**
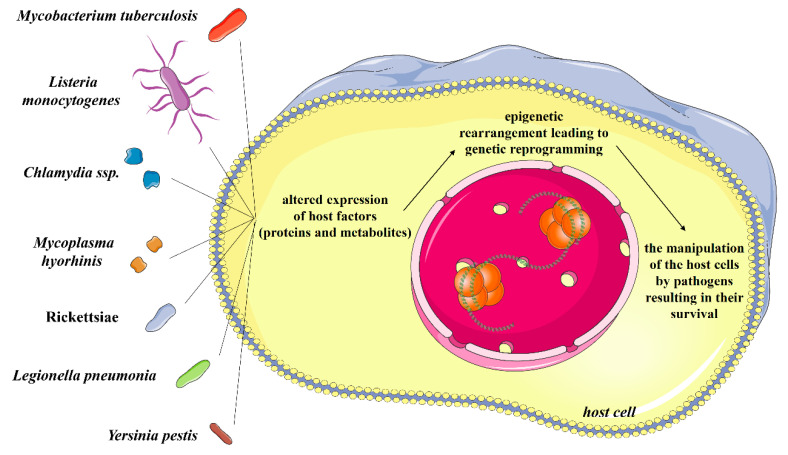
Strategies exploited by pathogens to modulate host epigenome.

Epigenetic regulation of the gene activity is a subject of deep and still-increasing interest. The appearance of an epigenetic trait defined as “a stably heritable phenotype resulting from changes in a chromosome without alterations in the DNA sequence” can be triggered by changes in the environment of the cell, precisely described and discussed by Berger et al. [1]. There is some evidence suggesting that certain microbial agents, e.g., *Helicobacter pylori* [2], *Porphyromonas gingivalis*, *Fusobacterium nucleatum* [3], *Streptococcus bovis*, *Chlamydia pneumoniae*, *Campylobacter rectus*, Epstein–Barr virus, hepatitis viruses, human papilloma virus, polyoma viruses, can contribute to the host epigenetic changes and are frequently associated with carcinogenesis [4]. In the context of host–pathogen interactions, microorganism trying to conquer the host would be regarded as co-called “epigenetor” (term proposed by Berger et al. [1])—a factor which descends from environment and triggers a cascade of events ultimately leading to the modulation of the host epigenome. The most common mechanisms by which epigenetics control changes in gene expression involve histone acetylation, histone deacetylation, histone methylation and DNA methylation [5,6,7]. However, these epigenetic modifications induced by the infectious agents in host cells are still not sufficiently explored. Possibly, these infectious agents have developed a wide variety of epigenetic regulatory mechanisms, through which they are able to effectively exploit the epigenome of the host for their own benefits (Figure 1) [8]. In this review, the main interest is focused on certain intracellular bacterial pathogens: *Mycobacterium tuberculosis*—an obligatory, aerobic bacillus still remaining one of the major global health problems since it is estimated that one fourth of global human population is infected with that pathogen [9,10] and *Listeria monocytogenes*—mainly transmitted through the consumption of contaminated food, causing listeriosis, a disease whose importance is not sufficiently recognized [11]. We have also compiled selected information regarding another four obligatory or facultative intracellular widespread bacteria of the genera *Chlamydia*, *Mycoplasma*, *Rickettsia*, *Yersinia* and *Legionella* [12,13,14]. Their strategies to modulate the host epigenome in order to overcome the host defense for their persistence are reviewed in the present study.

## 2. Mycobacterium Tuberculosis

*Mycobacterium tuberculosis*—the causative agent of tuberculosis (TB) in humans—is equipped with a broad spectrum of tools that allow it to survive and avoid the defense mechanisms of the host [15,16,17]. Ofthe most important mycobacterial abilities that enable the effective conquering of the host are some epifactors, through which this intracellular pathogen controls the expression of host genes at the chromatin level [18]. Yaseen et al. [19] described the mycobacterial protein Rv1988 (methyltransferase) responsible for dimethylation of arginine amino acid present specifically at the 42nd position within the core region of histone H3 (H3R42me2) in the host cell. This modification alters the expression of certain host genes, which benefits bacteria and supports the development of infection. It has been shown that this secretory protein is a product of only pathogenic mycobacteria (*M. tuberculosis*, *M. bovis*), while the other ones (*M. smegmatis*) do not express Rv1988. Following the tubercle bacilli entering the cell, released Rv1988 localizes with the chromatin in the host nucleus affecting the expression of genes, which are important for host defense, e.g., NOX1, NOX4, NOS2 and TRAF3 [18]. The first three ones are an important source of reactive oxygen species [20,21] and the last one, together with TRAF2, plays a pivotal role in cell type—and stimulus-specific production of type I IFN [22,23,24]. Interesting observations have been made on another secretory mycobacterial protein, namely Rv2966c. This 5-methylcytosine-specific DNA methyltransferase released by *M. tuberculosis* shows an ability to localize to the nucleus inside the infected mammalian cell. Rv2966c binds to specific DNA sequences and causes predominantly non-CpG methylation, and its activity is positively influenced by phosphorylation [25]. Similar to Rv1988, this protein can also interact with histone proteins, and probably both of them are the key elements of the first impact during infection hijacking the host defense control center by epigenetically altering its action [18]. As the cytosine methylation is rather commonly observed in mammalian cells and the Rv2966c shows dual nature characterized by the ability to be a dcm/dam-like prokaryotic DNA methyltransferase which binds to specific DNA sequences and by the ability to methylate cytosines that are not canonical dcm/dam sites, it could be said that at some level tubercle bacilli hijack the epigenomic control center of the host cell. Furthermore, this protein interacts with histone H3 and H4, and thus acts similarly to DNMT3L—one of the mammalian DNA methyltransferases also involved in nuclear reprogramming by binding to histone H3 and regulation of DNA methylation [25]. It is believed that during the course of TB infection, the manipulations regarding the regulation of host gene expression mediated by the pathogen are the key players, which allow the pathogen to survive in the host and to establish the disease [26,27]. Using the human inflammatory response methyl-profiler DNA methylation PCR arrays and the genome-wide CpG island microarray technique it was shown that during the mycobacterial infection (Beijing/W *M. tuberculosis* strains) of blood monocyte-derived macrophages, but not THP-1 cells, the enhancement in the hyper-methylation level occurred in the case of genes encoding IL17RA, IL15RA, IL6R and IL6ST, whereas the IL4R methylation was intensified in both types of macrophages [28]. Ip et al. [29] demonstrated that the methylation level of human macrophages differed depending on the stage of infection and was distinct between active TB, latent and healthy control cohorts. Significant DNA hypomethylation of FADD (as-associated protein with death domain) and IL17RA was noted in macrophages of patients with active TB disease. Assessing the percentage of hypermethylated CpG sites in the amplified regions of 24 inflammatory genes of THP-1 cells infected with two different groups of clinical *M. tuberculosis* strains (Beijing/W and non-Beijing/W strains), the significantly higher hypermethylation levels of the IL17RA, IL15RA, IL4R, IL6R, IL6ST genes as well asIL17RA, IL6R, IL6ST, respectively, were observed, when compared with uninfected macrophages [29]. Working with the bone marrow-derived macrophages it was found, that mycobacterial 19-kDa lipoprotein (Rv3763) inhibits IFN-γ-dependent CIITA (class II trans activator) expression [30], which has important effects on the expression of MHC-II and other molecules involved in antigen presentation [31]. It turned out that when the cells were infected by *M. tuberculosis*—or exposed to Rv3763—the IFN-γ increased acetylation of histones H3 and H4 at CIITA pIV locus was inhibited. It was also shown, that 19-kDa lipoprotein required TLR2 to mediate its inhibitory effect on IFN-γ-dependent chromatin remodeling of *MHC2TA* (the gene encoding CIITA). Furthermore, when macrophages were treated with inhibitors of MAPKs p38 or ERK, the inhibition of CIITA expression or IFN-γ-dependent histone acetylation of CIITA pIV by 19-kDa lipoprotein was not observed. Additionally, Rv3763 also inhibited IFN-γ-dependent recruitment of the ATP-dependent chromatin-remodeling protein, Brg1, to CIITA pIV. All of this suggests that *M. tuberculosis*-mediated disruption of IFN-γ-dependent chromatin rearrangement at *MHC2TA* leads to the inhibition of CIITA transcriptional activity [30]. Interesting data were provided by the study, which involved checking the macrophage DNA methylation status after infection with drug-susceptible and drug-resistant *M. tuberculosis* strains in the context of 22 genes involved in the TLR2 signaling pathway [32]. All of tested genes, except *Irak-2* and *Tbk-1*, were characterized by increased methylation levels when THP-1 macrophages were infected with XDR *M. tuberculosis* strains. In the case of infection with the susceptible strains, besides the increased methylation level of *Traf6* gene and hypomethylation of *Irak-2* gene, normal methylation of the other tested genes related to the TLR signaling pathway was observed, which may induce effective responses to infection. It was also shown that in all used drug-resistant mycobacterial strains the expression of *Rv1988* gene was elevated in comparison with susceptible strains and that was accompanied by the THP-1 methylation status in response to infection with those two groups of mycobacterial strains [32]. Apart from macrophages, also dendritic cells are recruited to the site of infection trying to limit the invasion of microorganisms [33,34]. The study regarding DNA methylation, gene expression and chromatin accessibility patterns showed that during the infection of human dendritic cells with *Mycobacterium tuberculosis*, a stable demethylation at enhancer elements was induced, including those associated with the H3K4me1 enhancer mark. It was found that immune-related transcription factors (TFs) such as NF-κB/Rel were recruited to enhancer elements before the observed losses in methylation, which suggests that DNA demethylation is mediated by TF binding to cis-acting elements [35]. Pacis et al. [36] indicated that demethylation observed in monocyte-derived dendritic cells after mycobacterial infection was associated with extensive epigenetic remodeling, including the gain of histone activation marks and increased chromatin accessibility. Interestingly, demethylated regions were a part of genomic regions that are characterized by increased levels of evolutionary conservation, which suggests their functional importance. It appeared that these regions were frequently present near genes playing an essential role in the regulation of transcription, signal transduction and cell apoptosis and included genes with the pivotal role in the immune response regulation: *CREB5*, *REL*, *NFKB1*, *IRF2*, *IRF4*, as well as *CD83* and *BCL2* [36].

## 3. Listeria

*Listeria* spp. is another interesting example of microorganism that can induce epigenetic and miRNA modifications in the host to modulate immune defense. *Listeria monocytogenes* is a Gram-positive facultative pathogen that causes the foodborne disease—listeriosis [37,38]. Following the entry into epithelial cells, *L. monocytogenes* is internalized into a vacuole. To escape from this niche by physically disrupting the vacuolar membrane it uses listeriolysin O (LLO) and phospholipase A and B (PlcA and PlcB) [39,40]. In general, *L. monocytogenes* is able to survive and replicate in the cytosol of host cells and modify host cell processes as well as organelles [41]. The cell membrane of *L. monocytogenes* involves actin assembly inducing protein (ActA), which interacts with the Arp2/3 (actin-related protein 2/3) complex and mediates actin polymerization generating an actin comet tail.This allows bacteria to spread from one cell to another [41,42]. How can *L. monocytogenes* manipulate host cell transcription and induce epigenetic modifications? One of the important classes of bacterial virulence factors are nucleomodulins that are secreted into the host cytoplasm and migrate to the nucleus to modulate gene expression. One of them is Listeria nuclear targeted protein A (LntA), which interacts with the chromatin repressing bromo adjacent homology domain-containing oneprotein (BAHD1) resulting in the upregulation of interferon-stimulated genes (ISG) in a type III interferon-dependent manner [43]. Moreover, *L. monocytogenes* also secrete cyclic small dinucleotide called cyclic di-AMP (c-di-AMP), which activates the stimulator of interferon genes protein (STING). The activation of innate immune pathways affects the stimulation of T cells resulting in the impaired clearance of the pathogen [41,44]. These various pathways upregulate, both directly and indirectly, the production of cytokines and other antibacterial proteins, contributing to the persistence of the infection.

The host cytoplasm contains nucleotide-binding oligomerization domain (Nod) proteins, which detect peptidoglycan in the bacterial cell wall. Nod is involved in the induction of the nuclear factor κB (NF-κB) activity, including activation of the receptor-interacting protein 2 (Rip2) and IκB kinase, as well as in the activation of caspases [45,46]. A study by Opitz et al. [46] showed that cytosol-localized *L. monocytogenes* induced NF-κB signaling and p38 MAPK (mitogen-activated protein kinases) phosphorylation via Nod 1. Moreover, they revealed that activation of Nod1was not essential for IFN-γ production, but it was crucial for IL-8 response [46].

As a key virulence factor, LLO has an ability not only to form pores in the cell membrane, but also to induce MAPK as well as calcium and NF-κB signaling. Moreover, LLO induces dephosphorylation of Ser10 on histones H3 and deacetylation of histones H4 [47,48]. Histone modifications are associated with transcriptional reprogramming of host genes, including upregulation in the expression of key immunity factors [47]. The production of CXCL2, one of the chemoattractant chemokines with proinflammatory function, has been found to be inhibited by LLO. It results in the impairment of polymorphonuclear cells recruitment, which weakens the innate immune response against *L. monocytogenes* [47,49].

MicroRNAs (miRNAs) are key modulators that affect the outcome of immune responses to infection at post-transcriptional levels [50]. Schnitger et al. [51] proved that the host genome-wide miRNA profile, including miR-155, miR-146a, miR-125a-3p/5p and miR-149, was altered during *L. monocytogenes* infection. miR-146a was found to regulate the activity of TNF receptor-associated factor 6 (TRAF6), IL-1 receptor-associated kinase 1 and 2 (IRAK1 and IRAK2), whereas miR-155 influenced IκB kinase epsilon (IKKε) [52]. Interestingly, these upregulations were not dependent on the hemolytic action of LLO, because using the Δ*hly* mutant strain did not abolish the activity of miRNAs [51]. Moreover, *L. monocytogenes* inhibited IFN-γ-induced autophagy of macrophages viamTOR (mammalian target of rapamycin) by Mir155 and Mir31 in the WNT signaling network and the activity of protein phosphatase 2 (PP2A) [53].

## 4. Chlamydia

Chlamydiae are obligate, intracellular, Gram-negative bacteria with a unique developmental cycle of replication consisting of extra- and intracellular forms. Within the family there are four species: *Chlamydia trachomatis*, *Chlamydophila pneumoniae*, *Chlamydophila psittaci* and *Chlamydophila pecorum* [54]. They are known as major pathogens of humans and animals; however, they are also present in a variety of environmental habitats. *C. trachomatis* and *C. pneumoniae*, the best-known species, are a cause of sexually transmitted diseases, ocular infections and atypical pneumonia [55,56].

It has been found that pathogenic chlamydiae have evolved diverse strategies to suppress the host cell response, mainly by targeting chromatin regulation via epigenetic modifications [57]. Analysis of the *C. trachomatis* genome identified a set gene encoding a protein with a domain similar to the eukaryotic SET domain, which is known to methylate lysine residues in the amino-terminal tail of histones [58,59]. This SET-containing protein in *C. trachomatis* called NUE has been shown to be secreted into the host cell during chlamydial infection, where it enters the nucleus and binds to host chromatin.

As a methyltransferase, the NUE is able to catalyze methylation of host histones H2B, H3 and H4. The homolog of NUE in *C. pneumoniae* was called cpnSET and its activity was determined against chlamydial histone-like proteins Hc1 and Hc2 as well as against mouse histones [60]. Apart from altering host chromatin structure by producing mimics of chromatin-modifying enzymes, chlamydiae is able to interfere with transcription by sequestration or deactivation of host transcription factors. One of the proteins recruited to the parasitophorous vacuole termed an inclusion is a zinc finger nuclear protein 23 (ZNF23), a repressor of cell division. It has been found that ZNF23 disappears from the host nucleus and cytoplasm and is incorporated into the lumen of the inclusion, along with acetyl-CoA binding protein ACBD6 [61]. It is suggested that the recruitment of ZNF23 to the inclusion may sequester the protein and prevent the activation of apoptotic pathways.

Nucleomodulins may not only bind DNA and chromatin factors, but also chromatin-anchoring factors. SinC, a protein secreted by *Chlamydophila psittaci* via a type III secretion system (T3SS) targets the inner membrane of the nucleus in infected cells and thus may control chromatin interaction with the nuclear lamina [62].

## 5. Mycoplasma

Mycoplasmas are the smallest self-replicating Gram-negative bacteria, which lack the genes coding for the cell wall. Instead of a cell wall, they possess a three-layered membrane, containing sterol, which is taken up from the environment. In humans, mycoplasmas are present frequently at mucosal surfaces of respiratory and urogenital tracts, mammary glands and joints [63]. Due to their frequent persistence as long-term asymptomatic infections they are likely to induce reprogramming of somatic cells and oncogenic cell transformation, resulting in dysregulation of cancer-specific genes. Mycoplasmas have been found to produce DNA methyltransferases responsible for the conversion of cytosine to 5-methylcytosine (5mC) in the context of CG-dinucleotides. Three DNA methyltransferases have been identified in *Mycoplasma hyorhinis*, an intracellular commensal that can shift to an opportunist pathogen. The Mhy1 and Mhy2methyltransferases, promote CG methylation, while the Mhy3 enzyme acts on GATC sites [64]. Chernov et al. [64] demonstrated that after translocation to the cell nucleus, the enzymes efficiently methylated the host genome at the DNA sequence sites free from pre-existing endogenous methylation, including those in a variety of cancer-associated genes. Yet, it remains to be proven that the host epigenome is reshaped in human cells naturally infected by *M. hyorhinis*.

## 6. Rickettsiae

*Rickettsia* spp. are obligate intracellular Gram-negative bacteria that require a vector for host transmission [65,66]. Rickettsiae are transmitted to humans by the bite of infected ticks and mites as well as by the feces of infected lice and fleas. Recent reports indicate that rickettsiae can be transmitted to human hosts even via mosquitoes [65]. Rickettsiae use surface cell antigens (sca0, sca1, sca5) and outer membrane proteins (OmpA and OmpB) to attach to host cell membrane followed by an active entry into the cell [67,68,69]. These bacteria are able to escape the phagocytic vacuole due to the activity of rickettsial phospholipase D and hemolysin [70]. The bacteria released into the cytoplasm of the host cell grow until they destroy the cell. In this process they use many host metabolic substrates, including nucleotides, enzymes for sugar metabolism as well as adenosine triphosphate (ATP) [71,72,73]. Rickettsiae utilize an intracellular actin-based motility (ABM) system to promote direct cell-to-cell spread as a result of propulsive polymerization of host cell actin, which allows them to move into pseudopodia that can be engulfed by neighboring cells afterwards [74,75]. Rickettsial diseases include spotted fever and typhus fever rickettsioses, however, disseminated infection may result in severe vasculitis and endothelial damage, clinically manifested cutaneous necrosis, pneumonitis, meningoencephalitis and multiorgan failure [65,76].

Intracellular pathogens have developed mechanisms that allow survival within hostile host environment. As a result of the bacterial influence on host chromatin modifications and defense gene transcription, intracellular pathogens cause dysregulation of host cell function leading to disease [37,77]. The cellular mechanisms responsible for epigenetic regulation includereversible post-translational histone modifications (acetylation, methylation and phosphorylation) and methylation of CpG dinucleotides in chromosomal DNA and noncoding RNA-mediated gene regulation [37,38,39,40,41,42,43,44,45,46,47,48,49,50,51,52,53,54,55,56,57,58,59,60,61,62,63,64,65,66,67,68,69,70,71,72,73,74,75,76,77,78,79].

Curto et al. [80] investigated the transcriptional responses in THP-1 macrophages infected with *R. conorii* and *R. montanensis*. They analyzed transcriptomic changes in 495 host genes as early as 1 h post-infection. The differentially expressed genes included the pro-inflammatory molecules TNFα, IL1β, as well as CCL20, CCL3L3, CCL3, CCL4L2, CXCL1, CXCL3 and CXCL8 that could shape the recruitment of immunocompetent cells to the site of infection. Moreover, they noticed changes in genes implicated in the modulation of the NF-κB pathway, including tumor necrosis factor, alpha-induced protein 3 (TNFAIP3) and NF-κB inhibitor zeta (NFKBIZ) [80]. Both of these molecules are known to inhibit NF-kappa B activation as well as TNF-mediated apoptosis [81,82,83]. Interestingly, only the pathogenic strain *R. conorii* enhanced the expression of mRNA for proteins involved in the JAK-STAT (Janus kinases—signal transducer and activator of transcription) signaling pathway. Considerable changes were observed in the genes for IL23A (interleukin 23 subunit A), OSM (oncostatin M) and SOCS3 (suppressor of cytokine signaling 3) [80]. All of these proteins play an important role in the control of immune responses. SOCS3 is a cytosolic suppressor of cytokine signaling of the gp130 family cytokine as well as γc family cytokine, which are involved in the maturation of Foxp3+CD25+ regulatory T cells [84]. The gene IL23A promotes the survival of T helper 17 (Th17) cells and formation of Th17 memory cells. Moreover, it enhances the production of proinflammatory molecules such as IL-1, IL-6, TNF-alpha, NOS-2 (nitric oxide synthase 2) [85]. As a pleiotropic cytokine, OSM regulates the expression of various proteases, protease inhibitors, acute phase proteins and intensifies the production of IL-6 [86,87,88]. Interestingly, *R. conorii* is able to modulate TNFα signaling in macrophages, which may influence inflammatory cell activation. In the study by Curto et al. [80] *R. conorii* infected cells responded with overproduction of TNFα following the treatment of THP-1 cells with LPS *E. coli*. This correlation was not observed in uninfected THP-1 cells or in the cells infected with non-pathogenic *R. montanensis*.

The rickettsial species can also modulate immunometabolism, although this process is still not well understood. Recent proteomic studies have revealed that rickettsiae modify the activity of host proteins involved in various metabolic processes [66]. Significant alterations were observed in glycolysis, pentose phosphate pathway (PPP), tricarboxylic acid (TCA) cycle, oxidative phosphorylation (OXPHOS), fatty acid metabolism and amino acid metabolism.

Rickettsial infection has been found to reduce the activity of enzymes involved in glycolysis and PPP, including glucose-6 phosphate isomerase, fructosebiphosphatase A, phosphoglycerate kinase 1, enolase 1, pyruvate kinase M1/2 and lactate dehydrogenase B [66]. These processes generate nicotinamide adenine dinucleotide phosphate (NADPH) and finally determine the synthesis of pentose sugars. The PPP is crucial for generation of M1 macrophages with high activity of NADPH oxidase and synthesis of ROS and NO [89,90]. On the contrary, in the *Rickettsiae*-treated THP-1 culture there was observed overproduction of citrate synthase, aconitase, isocitrate dehydrogenase 3 (IDH3A), fumarate hydratase (FH) isocitrate dehydrogenase 1 (IDH1) as well as malate dehydrogenase 1 and 2 (MDH 1 and 2) [66]. All of these enzymes are involved in TCA and OXPHOS cycles, which provide ATP to drive many processes in living cells [91].

Moreover, Curtoet al. [66] found that infection of THP-1 macrophages with pathogenic *Rickettsiae* resulted in the accumulation of proteins involved in the electron transport chain including cytochrome C1 (CYC1), ubiquinol-cytochrome c reductase core protein 1 and 2 (UQCRC1 and 2), cytochrome c oxidase subunit 4l1 (COX4l1) and cytochrome c oxidase subunit II (COX2).

Noteworthy is the fact that infection of THP-1 cells with *Rickettsiae* is associated with an increase in the expression of the host proteins responsible for lipid metabolic processes. The major role in this process is played by fatty acid synthase (FAS) involved in de novo fatty acid synthesis, which can be used in the life cycle of bacteria [66,92].

The differences observed in the activity of enzymes involved in metabolic pathways suggest reprogramming of defense genes in macrophages, induced by the rickettsial species.

## 7. Legionella

*Legionella* spp., a facultative intracellular Gram-negative bacterium, is an etiologic agent of the atypical pneumonia called the Legionnaires’ disease as well as the flulike infection known as the Pontiac fever [93]. The severe pulmonary form of legionellosis with the mortality of 15%–30% accounts for 1%–5% of all cases caused by the genus, whereas its milder form, in which the bacteria do not replicate inside alveolar macrophages, may occur even in more than 90% of the exposed population [94]. *Legionella pneumophila (L. pneumophila)*, responsible for most of the identified cases of legionellosis, is transmitted through inhalation of bacteria-containing aerosols from natural and artificial water sites [95].

*L. pneumophila* interferes with a wide range of host processes using them for its intracellular replication and survival [96]. The pathogen secretes a large number of effectors that enable it to avoid the host immune responses and modulate their function to its advantage. Most of them are secreted by the Dot/Icm type IV secretion system (T4SS) and, through their enzymatic activity, they act on different stages of the epigenetic regulation of eukaryotic gene expression. It has been shown that the molecules produced by *L. pneumophila* are able to ubiquitinate, phosphorylate, lipidate, glycosylate, AMPylate, de-AMPylate, phosphocholinate and dephosphocholinate various proteins of the host [97].

The pathogen modulates the ubiquitin signaling pathway by secreting molecules that mimic certain eukaryotic proteins. A LubX protein contains two *U-*box domains (*U-*box1 and *U-*box2) with similarity to eukaryotic E3 ubiquitin ligases, which can target cellular Clk1 (Cdc2-like kinase 1) during *L. pneumophila* infection [98]. Moreover, the LubX binds and polyubiquitinates another effector protein SidH, which leads to its proteasomal degradation in infected cells [99]. Apart from *U-*box containing protein, *L. pneumophila* also secretes some F-box-containing proteins (AnkB, LegU1) that interfere with ubiquitin signaling during infection of host cells. AnkB protein (*L. pneumophila* strain A100) containing the CAAX motif, promotes bacterial intracellular replication by recruitment of polyubiquitinated proteins on the *Legionella*-containing vacuole, whereas AnkB of *L. pneumophila* strain Paris lacking the CAAX motif modulates the ubiquitination of ParvB, a host protein present on focal adhesions and in lamellipodia [100]. The *L. pneumophila* LegU1 effector conferring E3 ubiquitin ligase activity has been found to target the host chaperone protein BAT3, a regulator of the endoplasmic reticulum stress response [101].

The process of protein phosphorylation plays a key role in the regulation of cell growth, differentiation or apoptosis. Two best-studied *L. pneumophila* serine/threonine protein kinases are LegK1 and LegK2 [97]. LegK1 phosphorylates the IκBα molecule, a member of the IκB proteins family, leading to the NF-κB activation and proinflammatory cytokines genes induction [102]. LegK2 kinase has been shown not to act in the NF-κB pathway, but it phosphorylates the host protein MBP [103].

Host glycosylation regulating cell signaling or gene transcription is another strategy used by *L. pneumophila* to promote its pathogenesis and survival. Three *Legionella* glycosyltransferases—Lgt1, Lgt2 and Lgt3—target eEF1A at Ser53 (eukaryotic translation elongation factor 1A), the most abundant protein synthesis factors and thereby inhibit the eukaryotic protein translation process [104]. An additional *L. pneumophila* glucosyltransferase SetA, introduced into target cells by a type IV secretion system, affects the intracellular host vesicle trafficking pathways [105].

AMPylation (adenylylation) and de-AMPylation, as well as phosphocholination and dephosphocholination, are other post-translational modifications of host proteins exploited by *L. pneumophila*. AMPylation is a process in which an adenosine monophosphate (AMP) moiety is covalently added to threonine, tyrosine of serine residues of a protein by using ATP [106]; *L. pneumophila* DrrA/SidM effector modulates adenylylation of Rab1b protein, the host regulatory protein recruited during the infection to the *Legionella*-containing vacuole [107,108,109]. De-AMPylation activity is assigned to SidD protein which mediates the removal of the AMP moiety from the modified Rab1. The *L. pneumophila* protein AnkX transfers a phosphocholine group from CDP-choline to a serine in the Rab1 and Rab35 GTPases [110], whereas the *Legionella* Lem3 (lpg0696) effector possessing an activity opposite to that of AnkX can remove the phosphocholine moiety from Rab1 [111].

## 8. Yersinia Pestis

*Yersinia pestis*, a facultative intracellular Gram-negative bacterium, is an etiological agent of plaguethat can infect humans via the oriental rat flea [112]. Thepathogen possesses a specialized type III secretion system to evade the immune responses of thehost [113]. One of the most important virulence factorsis the plague-protective antigen LcrV enabling the transport of *Yersinia* effector proteins named Yops across the host immune cell membrane, where they can exert cytotoxic and immunomodulatory effects [114]. Nucleomodulin YopM, a leucine-rich repeat protein, acts as a scaffolding protein facilitating the formation of a complex between two serine/threonine kinases—ribosomal S6 protein kinase 1 (RSK1) and protein kinase C-like 2 (PRK2) [115,116,117]. Recent data suggest that the YopM binds the DEAD-box helicase 3 (DDX3) to control the RSK1 in the nucleus leading to theincrease in transcription of immunosuppressive cytokines, such as IL-10 [118].

## 9. Epigenetic Modifications as Therapeutic Targets

Understanding the role of epigenetic reprogramming in the pathogenesis of infectious diseases is a challenging perspective. Elucidation of how chromatin remodeling influences the function of the innate immune system during the pathogen infection and following recovery, provides a scientific basis for arising new therapeutic opportunities. Recent data highlight a number of different pharmacological compounds used in vitro and in vivo that influence the immune response through macrophage tolerance and training. The development of site-specific histone deacetylase (HADC) inhibitors, bromodomain inhibitors, histone lysine methyltransferase (HKMT) inhibitors represent new tools in the modulation of histone post-translational modifications. The HDAC inhibitors were found to be a promising approach to purge the reservoir of persistent HIV infection [119], whereas inhibitors of bromodomain protein 4 (BRD4) exhibited substantial anti-viral activity against pseudorabies virus as well as a wide range of DNA and RNA viruses [120]. Furthermore, improved understanding of clustered regularly interspaced short palindromic repeats (CRISPR) editing system enabling targeted modification of the epigenomemay result in expanded applications in the field of infectious diseases providing clarification of host and microbe interactions and help in the prevention and treatment of infectious diseases [121].

## 10. Summary

Epigenetic reprogramming leading to the chromatin rearrangement and, in consequence, to alterations in the level of expression of certain factors/proteins is considered to be a crucial player determining the fate of cells/organisms. Epigenetic changes have been identified as the key events present in the course of the trained immunity phenomenon [122] as well as in the development of diseases characterized by flawed innate immune response [123]. However, these processes do not exhaust the list since it was proved that epigenetic modifications appeared to be involved in the strategy of survival of certain pathogens in the host (Table 1). The epigenetic interaction between pathogen and host is the object of still growing interest. This review presents how certain intracellular bacterial pathogens engage the variety of epifactors to evade host immunity and persist for a long time inside the host organism. They have been only partially identified and require further intensive investigation to understand precisely how pathogens hijack the host cell. This will allow the future development of new drug targets and biomarkers.

## Figures and Tables

**Table 1:** Bacterial modulators of host epigenetic changes.

	Bacterial Factor		Mechanism
*Mycobacterium tuberculosis* [19,25,30,31,32]			
Tuberculosis			
	Rv1988	methyltransferase	dimethylation of H3 (H3R42me2)
	Rv2966c	secretory protein	non-CpG methylation, methylation of H3 and H4
	Rv3763	lipoprotein	acetylation of histones H3 and H4, methylation of Traf6 gene, hypomethylation of Irak-2 gene
*Listeria monocytogenes* [41,43,44,45,46,47,48]			
Listeriosis			
	LntA	Listeria nuclear targeted protein A	activation of BAHD1
	c-di-AMP	cyclic small dinucleotide	activation of STING
	PG	peptidoglycan	Nod-dependent activation of NF-κB, p38 MAPK phosphorylation
	LLO	listeriolysin O	Induction of MAPK, dephosphorylation of H3, deacetylation of H4
*Chlamydia* spp. [58,59,60,62]			
Sexually transmitted diseases, ocular infections and atypical pneumonia			
*C. trachomatis*	NUE	SET-containing protein, methyltransferase	methylation of lysine in the amino-terminal tail of histones, methylation of H2B, H3 and H4
	cpnSET	methyltransferase	methylation of H3 and H4 and chlamydial histone H1-like proteins Hc1 and Hc2
*C. psittaci*	SinC	secretory protein	binding DNA and chromatin factors
*Mycoplasma hyorhinis* [64]			
Pneumonia, mild infections of respiratory system			
	Mhy	DNA methyltransferase	methylation of CG-dinucleotides
Rickettsiae [37,66,78,79,91,92]			
Spotted fever, typhus fever rickettsioses			
*R. conorii* *R. montanensis*			post-translational histone modifications, methylation of CpG dinucleotides in chromosomal DNA, modulation of host metabolic processes, accumulation of proteins involved in the electron transport chain, modulation of lipid metabolic processes
*Legionella pneumonia* [97,98,100,102,103,104,105,107,108,109,110,111]			
Atypical pneumonia (Legionnaires’ disease)			
	LubX		ligase Cdc2-like kinase 1, degradation SidH
	AnkB	F-box-containing protein	promotion of bacterial intracellular replication
	AnkB	F-box-containing protein	modulation of the ubiquitination of ParvB
	LegK1	kinase	activation of NF-κβ
	LegK2	kinase	phosphorylation of MBP
	Lgt1, Lgt2, Lgt3	glycosyltransferases	Inhibition of the eukaryotic protein translation process
	DrrA/SidM		modulation of adenylylation of Rab1b
	SidD		modulation of deadenylylation of Rab1
	AnkX, Lem3		modification of Rab1 and Rab35 GTPases
*Yersinia pestis* [114,115,116,117,118] Plague			
	Yops	Yersinia effector proteins	formation of a complex between two RSK1 and PRK2
			binding DDX3 and controlling RSK1
